# *Impatiens wutaishanensis* (Balsaminaceae), a new species from Southeast Yunnan, China

**DOI:** 10.3897/phytokeys.176.58825

**Published:** 2021-04-16

**Authors:** Rong-Li Liao, Lei Cai, Zhi-Yong Yu, Yue-Hua Wang, Wei-Bang Sun

**Affiliations:** 1 School of Life Sciences, Yunnan University, Kunming 650091, Yunnan, China Kunming Institute of Botany, Chinese Academy of Sciences Kunming China; 2 Yunnan Key Laboratory for Integrative Conservation of Plant Species with Extremely Small Populations / Key Laboratory for Plant Diversity and Biogeography of East Asia, Kunming Institute of Botany, Chinese Academy of Sciences, Kunming 650201, Yunnan, China Yunnan University Kunming China; 3 University of Chinese Academy of Sciences, Beijing 100049, China University of Chinese Academy of Sciences Beijing China; 4 Management Bureau of Jinping Fenshuiling National Nature Reserve, Jingping 661500, Yunnan, China Management Bureau of Jinping Fenshuiling National Nature Reserve Yunnan China

**Keywords:** China, Flora of Yunnan, *Impatiens parvisepala*, *Impatiens wutaishanensis*, morphology

## Abstract

*Impatiens wutaishanensis* R.L. Liao & Lei Cai, a new species from Southeast Yunnan, China, is here described and illustrated. This new species is most similar to *Impatiens parvisepala* S.X. Yu & Y.T. Hou in its racemose inflorescences, its four lateral sepals, the leaf arrangement, and in having yellow flowers. However, it differs in the height of the plants, the length of the petiole, the size and shape of the leaf blade, the shape of the spur, and the number of flowers in each inflorescence. A detailed description, color photographs, and a provisional IUCN red list assessment are provided, and its geographical distribution, ecology, and morphological relationship with relevant similar species are discussed.

## Introduction

The genus *Impatiens*Linnaeus (1753: 937) belongs to the family Balsaminaceae, and has a mainly Old World tropical and subtropical distribution, although a few species are found in the northern temperate regions of Europe, Russia and China as well as North America. There are over 1000 species known to the genus to date (Grey-Wilson 1980; Fischer 2004). Major biodiversity hotspots for *Impatiens* species include tropical Africa, Madagascar, southern India and Sri Lanka, eastern Himalayas, and Southeast Asia (Song et al. 2003; Yuan et al. 2004). In China, more than 270 species of *Impatiens* are recorded, of which over 240 are endemic to the country. Southwest China is, in the broad sense, part of the Southeast Asian area, and belongs to one of the biodiversity hotspots of the genus *Impatiens* with more than 200 species (Yuan et al. 2004; Xia et al. 2019a; Peng et al. 2020). The Chinese *Impatiens* species are mainly found in Southwest China’s Yunnan, Sichuan, Tibet, Guangxi and Guizhou Provinces (Chen 2001; Chen et al. 2007; Yu 2012; Zhang et al. 2013). In recent years, about 30 new species of *Impatiens* have been described from China, and a large proportion of these are found in Southwest China (e.g. Hou et al. 2011; Cai et al. 2015; Tan et al. 2015; Guo et al. 2016; Ding et al. 2016; Xia et al. 2019a, b; Lu et al. 2020). In 2015, during a botanical investigation in Jinping County, Southwest Yunnan Province, China, we collected some specimens of an unknown *Impatiens* species. After careful examination of the relevant specimens and literature of the genus *Impatiens* within the adjacent regions (Chen 2001; Chen et al. 2007; Hou et al. 2011; Shui et al. 2011; Yu 2012; Cai et al. 2015; Tan et al. 2015; Chinh et al.2015; Xia et al. 2019b), we concluded that the *Impatiens* species differed from previously reported or described taxa, and which we describe it here as a new species.

## Materials and methods

The material for this study was mainly collected during field surveys assessing the conservation status of the plants of Yunnan Province, China. Herbarium specimens were made carefully and dissected flowers were dried separately to enable examination and illustration in the laboratory. Additionally, flowers were preserved in formalin-acetic acid-alcohol (FAA) solution, and field notes were taken. The morphological characteristics of the new species were measured using a ruler and vernier calipers from both dried herbarium specimens and mature individuals of living plants in the field. Morphological features of the flowers were described and measured using a dissecting microscope.

Fresh pollen grains and leaf blades were collected from the living plants introduced from the field and cultivated at Kunming Botanical Garden (**KBG**) and loaded on the cryo-specimen holder and cryo-fixed in slush nitrogen (-210 °C), then sublimed and sputter-coated with Pt in a vacuum scanning electron microscopy (SEM) chamber at -140 °C. Morphological characters were observed, examined, and photographed with a cryo-SEM. The morphology of 30 pollen grains was measured and described according to terminology of pollen grains (Lu 1991; Janssens et al. 2012).

## Taxonomic treatment

### Impatiens wutaishanensis

Taxon classificationPlantaeEricalesBalsaminaceae

R.L. Liao & Lei Cai sp. nov.

7E11335A-DD95-5491-9F42-A0A0D34D00F1

urn:lsid:ipni.org:names:77216566-1

Figs 1 , 2

#### Type.

China, Yunnan Province, Jinping County, Maandi, alt. 1650 m a.s.l., 22°46'19.97"N, 103°28'29.78"E, 10 September 2016, Lei Cai & Z.Y. Yu CL16050 (holotype:1498854, KUN!; isotypes:1498855, KUN!).

#### Diagnosis.

This species is similar to *Impatiens parvisepala* S.X. Yu & Y.T. Hou (2011: 57) (Hou et al. 2011) in its racemose inflorescences, its alternate and aggregated or subverticillate at stem apex arrangement leaf, and its yellow flowers, but it can be distinguished by its conspicuous 0.5–2.4 cm long petiole (vs. sub-sessile or sessile), its shorter 10–35 cm high plants (vs. 35–60 cm high), its 3.5–12 × 1.5–4 cm elliptic to lanceolate-oblong leaf blades with cuneate bases (vs. 12–20 × 3.5–6 cm obovate or obovate-lanceolate blade with attenuate bases), its slightly incurved or narrowing to incurved spur (vs. nearly straight spur), and its racemose inflorescences with up to 22 flowers (vs. 6–8 flowers per inflorescence).

**Figure 1.**
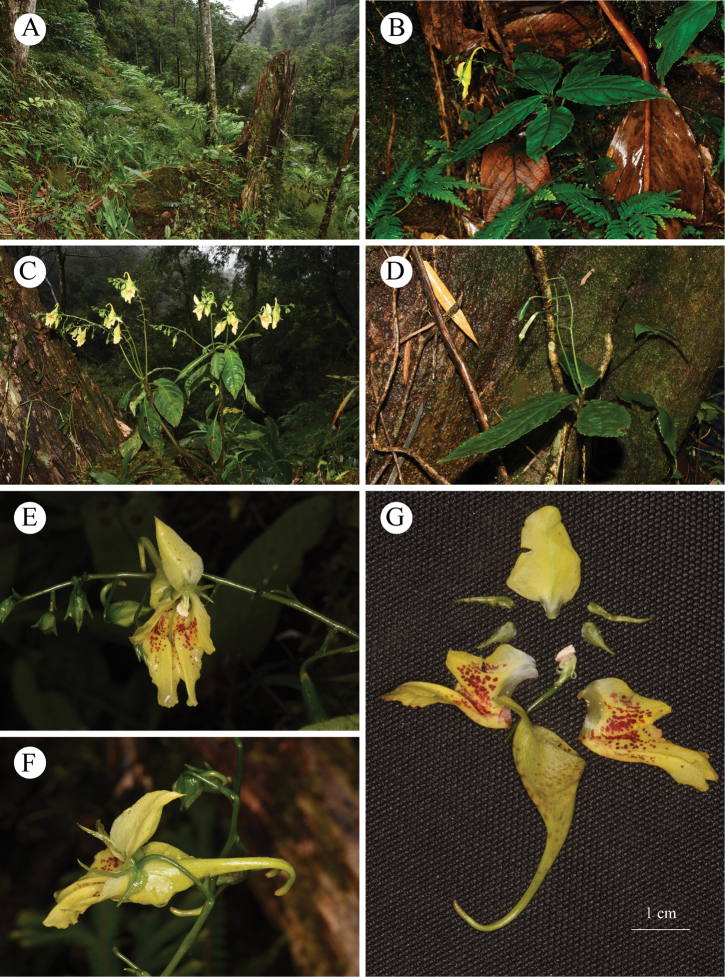
*Impatiens wutaishanensis* R.L. Liao & Lei Cai **A** habit **B** whole plant **C** inflorescence **D** capsule **E** flower in front view **F** flower in lateral view **G** different parts of the flower.

**Figure 2.**
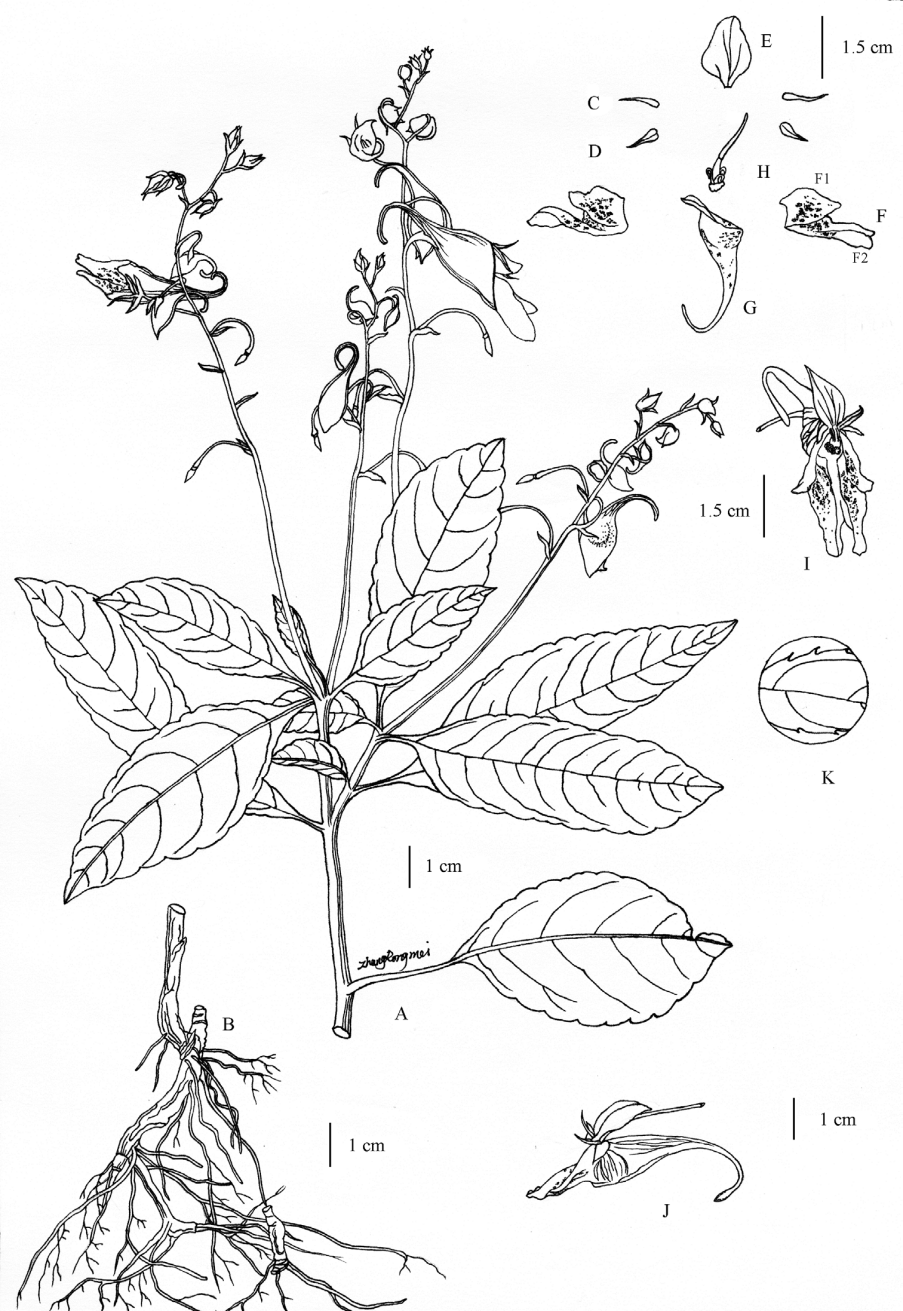
*Impatiens wutaishanensis* R.L. Liao & Lei Cai (drawn by R.M. Zhang) **A** habit **B** root **C** inner lateral sepal **D** outer lateral sepal **E** dorsal petal **F** lateral united petals (**F1**: upper petal **F2**: lower petal) **G** lower sepal **H** ovary and stamens **I** flower in front view **J** flower in lateral view **K** partial view of leaf margin.

#### Description.

Perennial herb, 10–35 cm tall, glabrous. Root-system shallow, with runners and adventitious roots. Stem fleshy, erect, simple, lower nodes swollen. Leaves simple, alternate, aggregated or subverticillate at stem apex; petiole 0.5–2.4 cm long, leaf blade 3.5–12 × 1.5–4 cm, elliptic, lanceolate, or lanceolate-oblong, base cuneate, margin roughly crenate, mucronulate, apex acuminate, adaxial surface dark green, abaxial surface pale green, lateral veins 4–8 pairs. Racemes in the upper leaf axils, 1–4, 4.2–28 cm long, erect, each with up to 22 flowers. Pedicels 0.6–2.5 cm long, bracteate at base. Bracts ca. 8 mm long, lanceolate to subulate, apex acute, base obtuse, margin entire. Flowers yellow. Lateral sepals 4, light green, the outer pair ca. 5–8 × 3–4 mm, ovate or obovate; the inner pair ca. 8–13 × 1–3 mm, sickle-shaped, obliquely lanceolate, apex acuminate or caudate. Lower sepal 1.5–2.2 × 1.2–2 cm excluding the spur, yellow to yellowish green with reddish patches, obliquely infundibuliform, base gradually constricted into a spur, spur 1.2–3 cm long, slightly incurved or narrowing to incurved, apex rostellate. Dorsal petal 1.3–2.3 × 0.7–1.6 cm, yellow with nearly transparent base, obovate to ovate, apex acuminate, with an inconspicuous dorsal crest, base truncate or cuneate. Lateral united petals ca. 1.6–3 cm long, yellow with nearly transparent base and reddish patches; the upper petals ca. 1.5–2.3 × 0.7–1 cm, oblong; the lower petal ca.1.2–1.8 × 0.6–0.8 cm, reniform. Stamens 5, filaments linear, ca. 3 mm long, anthers obtuse. Ovary fusiform, slightly curved. Placentation axile with four locules. Capsule (immature) clavate, 2–2.5 cm long.

#### Pollen morphology.

**Figure 3.**
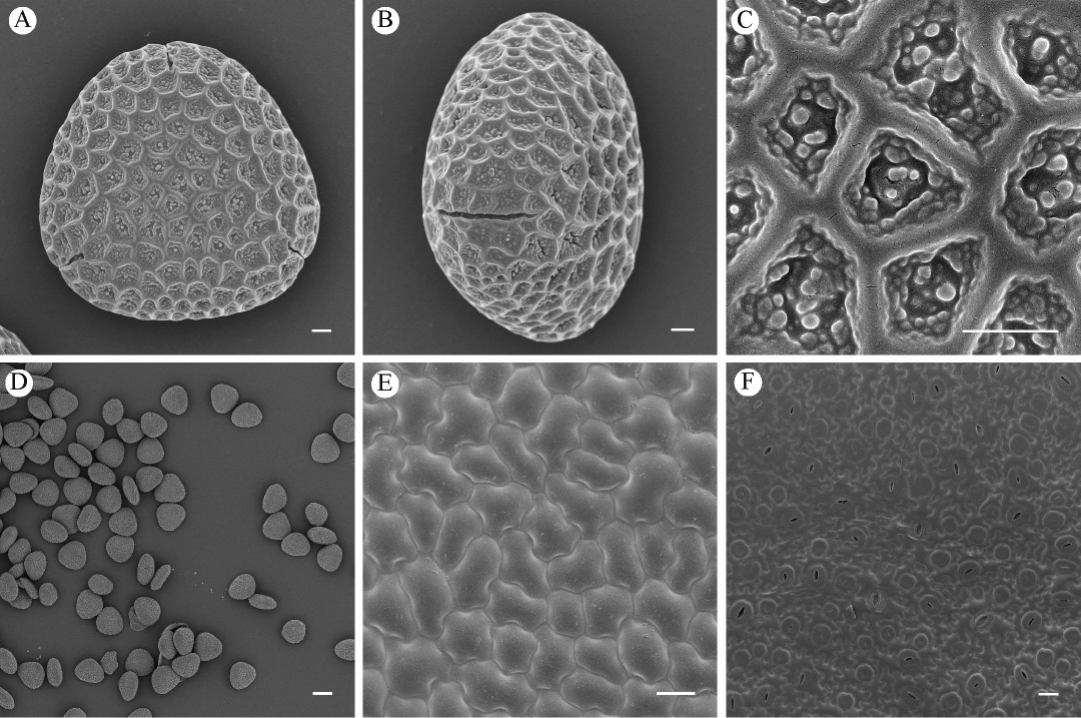
Micromorphology of *Impatiens wutaishanensis* R.L. Liao & Lei Cai under cryo-SEM**A–D** pollen **A** polar view **B** equatorial view **C** partial view **D** total view **E–F** leaf surface **E** adaxial epidermis **F** abaxial epidermis. Scale bar: 2 μm (**A, B**); 1 μm (**C**); 20 μm (**D–F**).

Pollen grains triangular-round with three equal sides in polar view, and the equatorial view is elliptic, long-elliptic, P×E=16.56±1.78 (14.51–21.73)× 30.00±0.98 (28.35–32.11) μm. 3-colpate, linear, the entire surface is covered with reticulate ornamentation, granules in lumina (Fig. 3).

#### Leaf surface micromorphology.

The anticlinal walls of the epidermal cells on the adaxial surfaces are straight, while those of the abaxial surface are sinuate. The stomata only appeared on the abaxial surface of the leaf, the shape was anomocytic and the outline of the guard cells was suborbiculate (Fig. 3).

#### Phenology.

This new species was observed flowering from August to November, and fruiting from September to December.

#### Distribution.

**Figure 4.**
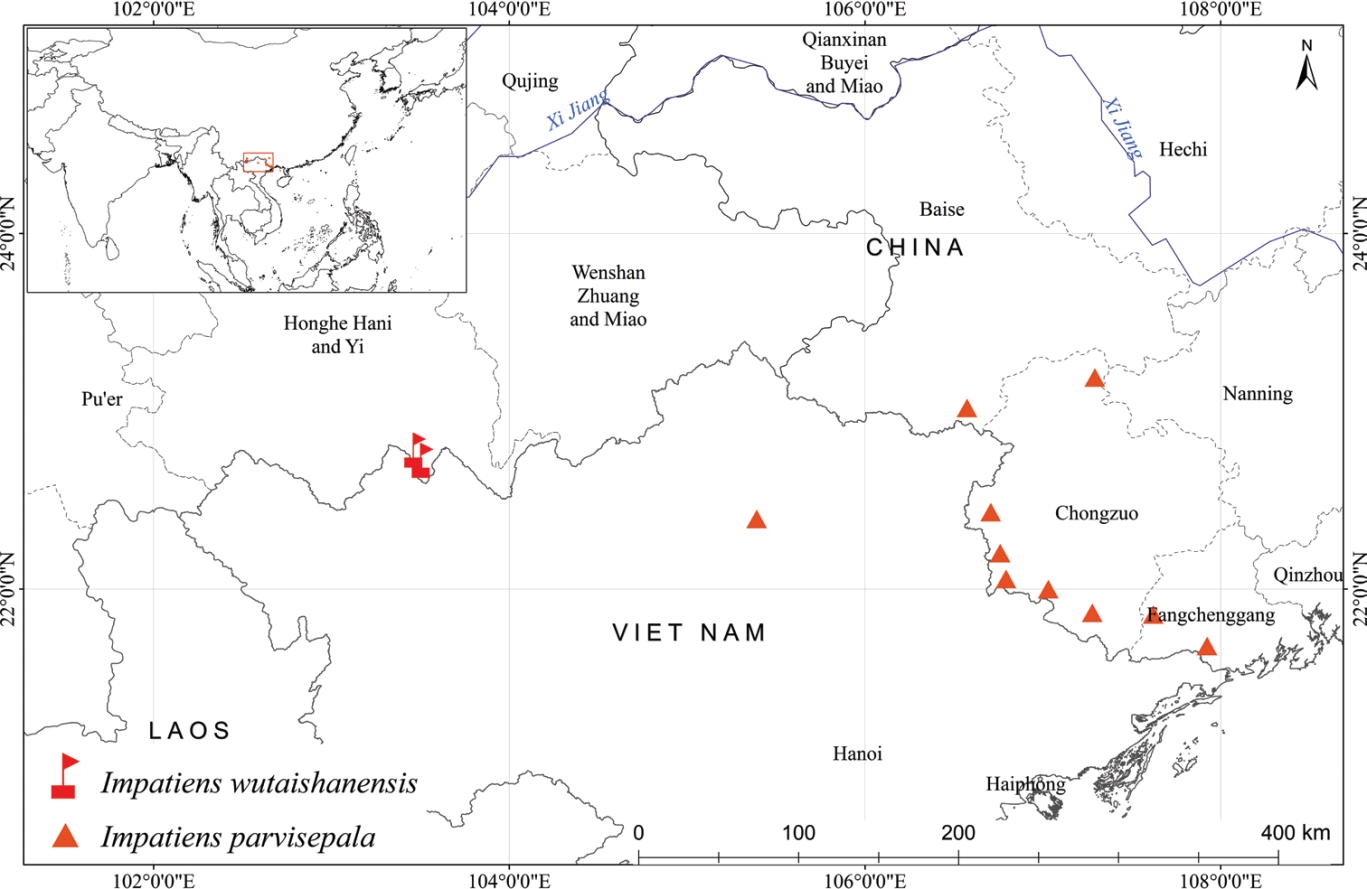
Distribution of *Impatiens wutaishanensis* R.L. Liao & Lei Cai and *I. parvisepala* S.X. Yu & Y.T. Hou.

This species is currently known from only two small subpopulations less than 10 km away (Shidong and Biaoshuiyan) in Jinping County of Southeast Yunnan, China (Fig. 4). The distribution area is very close to the border between China and Vietnam. We assume that this species should be distributed in Vietnam due to its similar habitat and proximity to the type locality, which will be verified by future investigation.

#### Ecology.

This new species has a runner with adventitious root produced from the nodes and was collected growing in the understory of forest at an elevation of 1300–1650 m. In the subpopulation in Shidong, the plants found coexist with the cultivated *Amomum tsaoko* Crevost & Lemarie (Zingiberaceae). In the subpopulation in Biaoshuiyan, the plants were found beside the artificial trail in a scenic area. The main accompanying species were *Begonia* Linn. sp. (Begoniaceae), *Didymocarpus purpureobracteatus* W.W. Smith (Gesneriaceae), *Epigeneium amplum* (Lindl.) Summerh. (Orchidaceae), *Hedychium villosum* Wall. (Zingiberaceae), *Polygala fallax* Hemsl. (Polygalaceae) and *Vaccinium* Linn. sp. (Ericaceae).

#### Conservation status.

This species is currently known only from Jinping County, Yunnan, China with one type locality and a subpopulation. The Extent of Occurrence (EOO) is less than 100 km^2^ and the known Area of Occupancy (AOO) is less than 15 km^2^. The conservation status can be evaluated as Vulnerable (VU) D2 based on IUCN Red List Categories and Criteria (IUCN 2019). About 350 and 200 individuals were known in the two subpopulations of Biaoshuiyan and Shidong, respectively, and both are exposed to human disturbance. Therefore, we assess this species as a Plant Species with Extremely Small Populations (PSESP) (Yang et al. 2020).

#### Etymology.

The specific epithet ‘*wutaishanensis*’ refers to the type locality where the new species was found, located in the Wutaishan area of Jinping Fenshuiling National Nature Reserve, Jinping County, Southeast Yunnan, China. The Chinese name is given as “五台山凤仙花”.

#### Additional specimens examined.

Paratypes. China. Yunnan Province: Jinping County, Maandi Town, Shidong. 22°46'19.97"N, 103°28'29.78"E, 1650 m a.s.l., 5 August 2015, Lei Cai et al., CL155 (KUN!); The same locality, 2 August 2020, R.L. Liao & X.Y. Li, LRL202008003 (KUN!); Yunnan Province: Jinping County, Maandi Town, Biaoshuiyan, 22°43'56.61"N, 103°30'36.58"E, 1311 m a.s.l., 2 August 2020, R.L. Liao & X.Y. Li, LRL202008001 (KUN!)

#### Discussion.

The new species is most similar to *Impatiens parvisepala* in its racemose inflorescence, its yellow flowers with four lateral sepals. However, *I. wutaishanensis* is usually a shorter plant and its petiolate leaf blades are smaller and elliptic, lanceolate, or lanceolate-oblong. Its nectar spur is slightly incurved or narrowing to an incurved spur, and each inflorescence may have up to 22 flowers (Figs 1, 2). In contrast, the plants of *I. parvisepala* are taller, the sessile or subsessile leaf blades are larger and obovate or obovate-lanceolate, the spur is nearly straight and the number of flowers per inflorescence is 6–8 (Hou et al. 2011; Son et al. 2015). The distribution of these two species is geographically isolated from each other: *Impatiens wutaishanensis*, is confined to Southeast Yunnan Province with two subpopulations, where the *I. parvisepala* is recorded in western Guangxi and northern Vietnam (Fig. 4).

In order to illustrate the morphological circumscription of the new species, we compare the new species with four species with similar morphological characters in Table 1: *Impatiens apalophylla* Hook. f. (1908:243), *I. clavigera* Hook. f. (1908:2863), *I. parvisepala* and *I. tianlinensis* S.X. Yu & L.J. Zhang (2015: 253) (Zeng et al. 2015).

**Table 1.** Comparison of morphological characters in *Impatiens wutaishanensis* (n=30 for the measurements), *I. parvisepala* (data from Hou et al. 2011 and Son et al. 2015), *I. apalophylla* (data from Hooker 1908a and Chen et al. 2007), *I. clavigera* (data from Hooker 1909b, Hou et al. 2011 and Tan et al. 2015,) and *I. tianlinensis* (data from Zeng et al. 2015).

Character	*I. wutaishanensis*	*I. parvisepala*	*I. apalophylla*	*I. clavigera*	*I. tianlinensis*
Plant height	10–35 (16.71±5.30) cm	35–60 cm	30–60 cm	50–60 cm	50–80 cm
Length of petiole	0.5–2.4 (1.02±0.44) cm	sub-sessile or sessile	2–4 cm	1–2 cm	(0.5–) 1–2 cm
Shape of leaf blade	elliptic, lanceolate, or lanceolate-oblong	obovate or obovate-lanceolate	oblong-ovate or oblong-oblanceolate	obovate or oblanceolate	obovate to oblanceolate
Size of leaf	3.5–12 (7.53±1.86) × 1.5–4 (2.90±0.79) cm	12–20 × 3.5–6 cm	10–22 × 4–8 cm	8–15 (–18) × 3.5–5 cm	10–15 (–18) × 5–8 cm
Length of peduncle	4–28 (13.00±6.71) cm	15–17 cm	7–15 cm	8–10 cm	10–15 cm,
No. of flowers in an inflorescence	up to 22 flowers	6–8 flowers	4–10 flowers	5–9 flowers	3–5 (–7) flowers
Shape and length of spur	slightly incurved or incurved, gradually constricted, 1.2–3 (2.06±0.45) cm long	nearly straight, gradually constricted, 2–2.5 cm long	incurved, abruptly elongated, 2–2.5 cm long	incurved, abruptly narrowed, 5–6 mm long	involuted, abruptly constricted, 1–1.5 cm long
Bracts	lanceolate to subulate, persistent	lanceolate or subulate, persistent	invisible	ovate, caducous	ovate, deciduous
Lower sepal	obliquely infundibuliform, with reddish patches, 1.2–2.8 (1.94±0.38) × 0.8–2.2 (1.38±0.37) cm	obliquely infundibuliform, with reddish patches, 1.8–2 × 2.5–3 cm	narrowly funnel-shaped, with reddish patches, 3 cm	narrowly funnel-shaped, absent patch, 2 × 3 cm	slightly narrowly funnel-shaped, with reddish patches, 2.5–3.5 cm
Dorsal petal	ovate, 1.3–2.3 (1.80±0.28) × 0.7–1.6 (1.15±0.26) cm	obovate, 1.8–2.2 × 1.5–2 cm	ellipticum, 1–4 cm	obovate, ca. 2 cm	ovate, 10–12 × 7–9 mm
Lateral united petals	1.6–3 cm long, with reddish patches	2.8–3 cm long, with reddish patches	2–3 cm long, with apparent reddish patches	2–6 cm long, absent patch	2–2.5 cm long, with reddish patches
Pollen grains	30.00±0.98 (28.35–32.11) × 16.56±1.78 (14.51–21.73) μm	28.84 (27.98–30.17) × 20.57 (19.70–21.63) μm	31.85 (30.96–32.73) × 5.57 (5.13–6.75) μm	Missing data	30.13 (29.62–30.47) × 12.95 (12.68–13.54) μm

## Supplementary Material

XML Treatment for Impatiens wutaishanensis
